# Fisetin in Cancer: Attributes, Developmental Aspects, and Nanotherapeutics

**DOI:** 10.3390/ph16020196

**Published:** 2023-01-28

**Authors:** Rachna M. Kumar, Hitesh Kumar, Tanvi Bhatt, Rupshee Jain, Kanan Panchal, Akash Chaurasiya, Vikas Jain

**Affiliations:** 1 Department of Pharmaceutics, JSS College of Pharmacy, JSS Academy of Higher Education & Research, Mysuru 570015, India; 2 Department of Pharmaceutical Chemistry, JSS College of Pharmacy, JSS Academy of Higher Education and Research, Mysuru 570015, India; 3 Department of Pharmacy, Birla Institute of Technology and Science, Pilani Hyderabad, Telangana 500078, India

**Keywords:** cancer, fisetin, flavonoids, nanocarrier, phytoconstituent

## Abstract

Cancer is one of the major causes of mortality, globally. Cancerous cells invade normal cells and metastasize to distant sites with the help of the lymphatic system. There are several mechanisms involved in the development and progression of cancer. Several treatment strategies including the use of phytoconstituents have evolved and been practiced for better therapeutic outcomes against cancer. Fisetin is one such naturally derived flavone that offers numerous pharmacological benefits, i.e., antioxidant, anti-inflammatory, antiangiogenic, and anticancer properties. It inhibits the rapid growth, invasiveness, and metastasis of tumors by hindering the multiplication of cancer cells, and prompts apoptosis by avoiding cell division related to actuation of caspase-9 and caspase-8. However, its poor bioavailability associated with its extreme hydrophobicity hampers its clinical utility. The issues related to fisetin delivery can be addressed by adapting to the developmental aspects of nanomedicines, such as formulating it into lipid or polymer-based systems, including nanocochleates and liposomes. This review aims to provide in-depth information regarding fisetin as a potential candidate for anticancer therapy, its properties and various formulation strategies.

## 1. Introduction

Cancer is one of the most challenging diseases that has a major global health impact. In 2020, there were 19.3 million new instances of cancer and 10 million cancer-related deaths, worldwide [1]. The most common type of cancer in both men and women is lung cancer, which is followed by breast, prostate, and colorectal cancer, in terms of incidence; and colorectal, stomach, and liver cancer in terms of death [2].

A series of genetic changes that affect biological functions, such as cell division, proliferation, transcription, and gene expression, lead to cancer. Cancer cells become somewhat self-sufficient, which causes uncontrollable cell proliferation and division, resulting in the spread of malignant cells throughout the body. Cancer cells continue to divide actively because they lack the ability to influence a cell’s homeostatic system. Instead, they produce oncogenic proteins that mimic the growth signals seen in healthy cells [3]. Carcinogenesis is the process by which healthy cells transform into self-sufficient cancer cells. These processes may take place at the genome, epigenome, or cellular levels, among other levels [4].

Oncogenic transition causes malignant cells to become independent of growth cues, resulting in uncontrolled growth. As a result, they generate their own signals and transmit them via a process known as the signal transduction pathway to other signaling proteins [5]. The intracellular signaling molecules, receptors, and extracellular growth signals, all exhibit significant alterations. The proto-oncogenes that produce proteins to promote cell division along with signaling molecules promote the expression of oncogenes, and encode for multiple factors that promote tumor progression [6,7].

**Figure 1:**
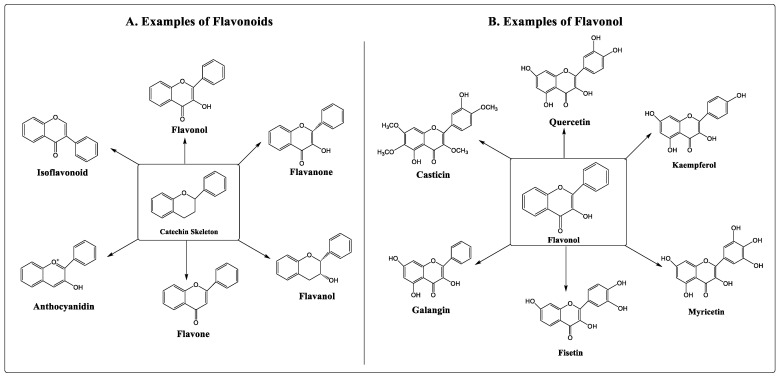
Types and examples of flavonoids (**A**) and flavonols (**B**).

Numerous studies have shown that flavonoids can prevent the development of cancer [8,9]. Flavonoids are secondary metabolites classified under the group of polyphenolic compounds. These naturally existing compounds are commonly dispersed in a plant’s leaf, stem, and root [10,11]. They exhibit remarkable outcomes in the human body, including anti-allergic, antioxidant, anti-inflammatory, and antiviral activities [12,13]. Moreover, flavonoids have been found to inhibit the proliferation, growth, and metastasis of breast cancer in vitro as well as in animal models [14,15]. Its use as an anti-carcinogenic agent is attributed to its antioxidant and anti-inflammatory properties [16]. Flavonoids interfere with multiple signal transduction pathways that occur during carcinogenesis, thereby reducing proliferation, angiogenesis, and metastasis, or increasing apoptosis, making them potential anticancer agents. However, its dose-dependent pharmacokinetics and low-dose potency contribute to some therapeutic obstacles [17,18]. Based on their structure, flavonoids are classified into six categories, viz., flavonol, flavanone, flavanol, flavone, anthocyanidin, and isoflavonoid. Quercetin, kaempferol, fisetin, myricetin, galangin, and casticin are flavonols with anticancer properties (Figure 1).

**Figure 2:**
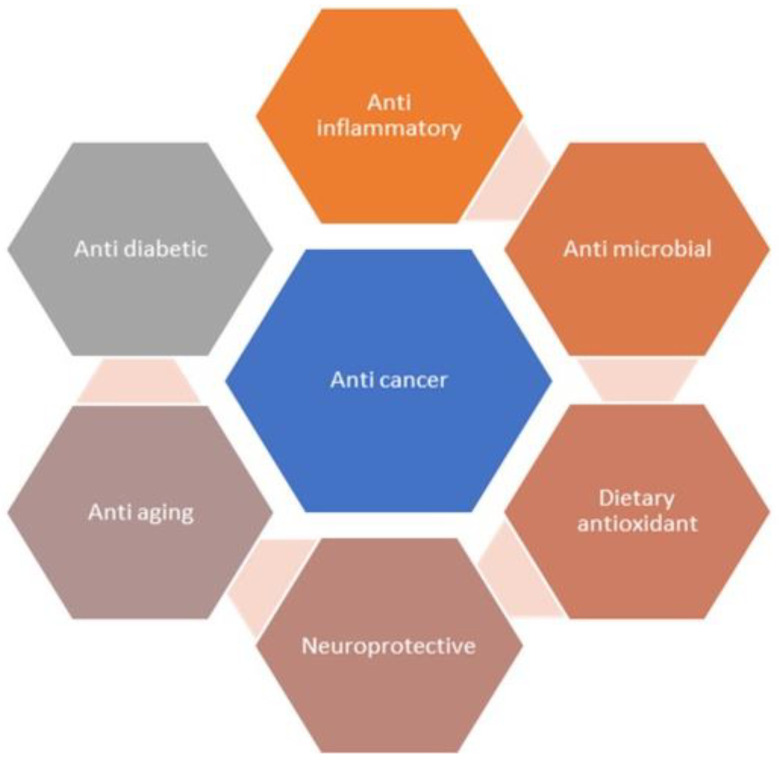
Pharmacological effects of fisetin.

Fisetin is a flavonoid naturally occurring in various plants that possesses anticancer activity [10,19]. It has the power to stop cancers from growing quickly, becoming invasive, and spreading to multiple tissues. Similar outcomes of fisetin were reported in various preclinical studies with melanoma, and with pancreatic, prostate, and colorectal cancer [20,21,22]. Fisetin chemically is a 3,3′,4′,7-tetrahydroxyflavone, and offers multiple pharmacological benefits, which include antioxidant, anti-inflammatory, antiangiogenic, and anticancer activities (Figure 2) [23,24,25,26]. Moreover, fisetin also inhibits tumor proliferation by repressing tumor mass multiplication, invasion, migration, and autophagy. It also promotes cell cycle averting and cell death in many types of cancers, which include prostate, breast, lung, bladder, melanoma, and hepatocellular cancers, and nasopharyngeal carcinoma [27,28,29].

In this review, we focus on fisetin as a potential candidate for anticancer therapy. We also discuss its physicochemical properties, pharmacological action, pharmacokinetics, bioavailability profile, and toxicity. This review also summarizes various formulations and drug delivery strategies employed to enhance the therapeutic efficacy of fisetin. Patents on fisetin that are currently available are discussed as well.

## 2. Physicochemical Properties and Synthesis of Fisetin

Fisetin has a structure of diphenylpropane, containing two aromatic rings. Its molecular formula is C_15_H_10_O_6_, and its molecular mass is 286.239 g/mol. With a density of 1.688 g/mL, fisetin melts at 330 °C, indicating its crystalline nature. As a result of this property, it exhibits lower solubility in water and a positive log P of 0.151 mg/mL and 1.81, respectively [30]. It is essentially insoluble in benzene, chloroform, ether, and petroleum ether, in addition to water. However, it is soluble in organic solvents such as alcohol, acetone, acetic acid, DMF, and DMSO [31]. Quantitatively, the solubilities of fisetin in ethanol, DMSO, and DMF are approximately 5 mg/mL and 30 mg/mL, respectively [31]. The strong acidic and basic pKa values of fisetin are 6.32 and −3.9, respectively [30,32]. Thus, it also exhibits solubility in a solution of fixed alkali hydroxide [31]. The physicochemical properties of fisetin are summarized in Table 1. The concentration of fisetin in plant sources is measured by freeze-drying the plants, which is the acid hydrolyzed product of the parent glycosides. The daily uptake of fisetin was calculated to be an average of 0.4 milligrams. The highest amount of fisetin detected in strawberries was found to be approximately 160 µg/g, followed by apple and persimmon, which were approximately 26.9 µg/g and 10.5 µg/g, respectively [33].

Fisetin is available naturally in various plant sources; however, it was first extracted from venetian sumach (*Rhus cotinus* L.) in 1833. Fisetin is also extracted from strawberries and mulberry leaves, using methanol extraction followed by liquid-liquid extraction [34,35]. The extract of persimmon fruit also contains fisetin as one of its active constituents, which was extracted using a number of methods, and quantified using liquid chromatography [36].

Later, the chemical characteristics and structure of fisetin were elucidated by S. Kostanecki in 1890s. The first synthesis of fisetin was performed in 1904, which involved the preparation of partially protected chalcones, further cyclized to flavanone under acidic conditions. The stable structure of fisetin was synthesized through several steps, such as oxidation, hydrolysis, and demethylations of chalcone and flavanone counterparts [37,38].

## 3. Mechanism of Action

Fisetin acts on different stages of cancer, thus providing different routes of inhibition. It affects the cell cycle and thereby cell proliferation, microtubule assembly, cell migration and invasion, epithelial to mesenchymal transition (EMT), and cell death [39]. It helps in the downregulation of approximately 27 genes involved in critical functions of the G2/M phase. It also exhibits affinity and specificity for significant cell cycle regulatory molecules, such as CDK6. Upon co-crystallization with CDK6, it was found that fisetin successfully inhibited kinase activity, which is one of the major drivers in cancer [40,41]. Thus, it regulates cell survival and growth as well as cell proliferation by controlling various signaling mechanisms [42]. The cell death caused by fisetin is possibly due to the induction of apoptosis by fisetin or other signaling molecules and reactive oxygen species (ROS). Moreover, fisetin also increases the sensitivity of cells toward apoptosis, making cancerous cells more susceptible to its oncogenic activity [39]. An additional mechanism by which fisetin inhibits cancer growth is through its action inside the nucleus of tumor cells. The development of breast cancer is significantly influenced by RNA polymerase I (RNA Pol I). Fisetin has been seen to penetrate the nucleolus, where it interferes with ribosomal RNA biogenesis. Fisetin was found to have a 50–70% reduction in nascent rRNA synthesis, and a 30–60% downregulation of RNA Pol I transcriptional activity, in a study that examined the nuclear activity of fisetin. Thus, rRNA biogenesis is a potential target for treating breast cancers and other metastatic tumors [43]. At the molecular level, the effect of fisetin is significantly mediated through activation and modulation of the SEMA3E, CDKN1A, GADD45A, and GADD45B genes of signaling pathways, and by the downregulation of the CCNB1, CCNB2, KIF20A, and TOP2A genes.

Without influencing the growth of normal cells, fisetin has the capability to hinder the formation of colonies and inhibit the multiplication of cancer cells. Moreover, fisetin restricts the multiplication of EGFR 2-overexpressing SK-BR-3 breast tumor masses, and breast cancer cells with estrogen receptors. It prompts apoptosis by avoiding cancer cell division related to the actuation of caspase-9 and caspase-8 and permeabilization of the mitochondrial membrane, followed by the splitting of poly (ADP-ribose) polymerase-1. The decreased destruction of tumors in the presence of pancaspase inhibitors such BOC-D-FMK and Z-VAD-FMK was evidence of caspase-dependent apoptosis. When tumor cells are exposed to drugs during the G2/M phase, there is a decrease in histone H3 phosphorylation at serine 10, which suggests that drug-induced death results from Aurora B kinase inhibition [27]. Aurora B kinase is directly inhibited due to the antiproliferative effect of fisetin, which causes the initiation of apoptosis in various tumor cell lines, and constrains exit from mitosis [44]. Additionally, fisetin inhibits cancer metastasis by reducing the expressions of nuclear factor-kB (NF-kB)-modulated metastatic proteins in a variety of tumor cell types, including vascular endothelial growth factor (VEGF) and matrix metalloproteinase-9 (MMP) [45,46]. Fisetin targets the NF-B- and mitogen-activated protein kinase signaling pathways to reduce the invasiveness of malignant melanoma [47]. 

Fisetin induces apoptosis in caspase-3-deficient MCF-7 breast cancer cells by rupturing the plasma membrane, depolarizing mitochondria, cleaving PARP, and activating caspase-7, -8, and -9. Moreover, autophagy inhibition promoted MCF-7 cell death [28,48], and was recently reported to weaken 12-O-tetradecanoylphorbol-13-acetate-induced obtrusiveness of MCF-7 and hepatic stellate cells [49,50]. Fisetin is a bioactive flavonol molecule that can easily penetrate the cell membrane due to its hydrophobic nature [51,52], reducing the generation of inflammatory cytokines and reactive oxygen species (ROS) in microglial cells, as well as inflammation-related microglial activation [53]. Fisetin has an antioxidative property that helps lower oxidative stress, leading to neuronal death in the case of stroke and arteriosclerosis [54]. Recent studies have likewise shown that fisetin exerts an antiproliferative effect against several cancer types [55]. In addition, evidence proves that fisetin is more targeted to tumor cells than to normal cells.

**Figure 3:**
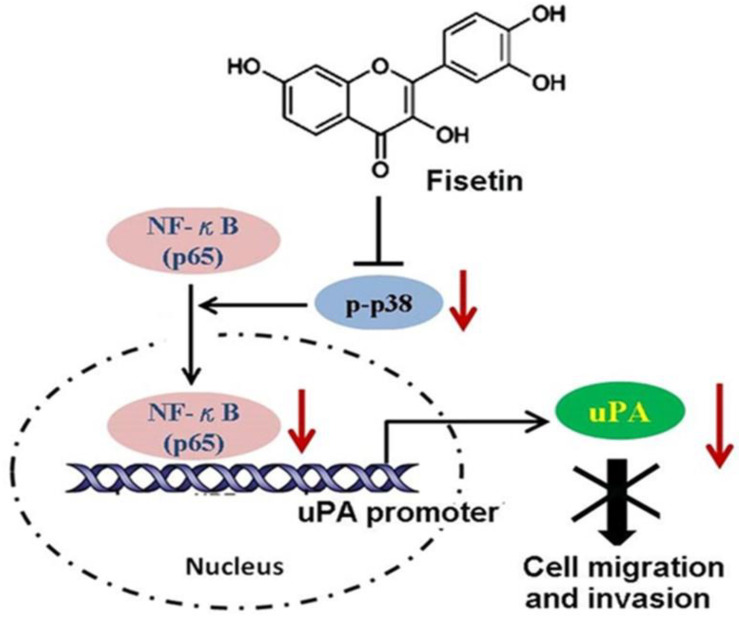
Fisetin inhibits the migration and invasion of cancer cells. Fisetin inhibits the phosphorylation of p38 MAPK, and impairs translocation of NF-κB to the nucleus. The decreased NF-κB in the nucleus reduces its binding to the promoter of the uPA gene, and results in repressing the expression and activity of uPA, thereby disrupting the migratory and invasive ability of cancer cells. Adapted from [57]. Copyright: © 2013.

The in vitro and in vivo reports provide information implying that fisetin has antiproliferative properties against various types of cancer [21]. Perhaps fisetin lowers angiogenesis, consequently suppressing tumor multiplication by urokinase plasminogen activator (uPA) inhibition (Figure 3) [56,57]. The effect of 17 structure-related flavonoids was evaluated in a screening study, where fisetin was found to be a powerful matrix metalloproteinase (MMP)-1 inhibitor, which has a crucial role in cancer progression, and is a prime enzyme in the destruction of the extracellular matrix [58].

**Figure 4:**
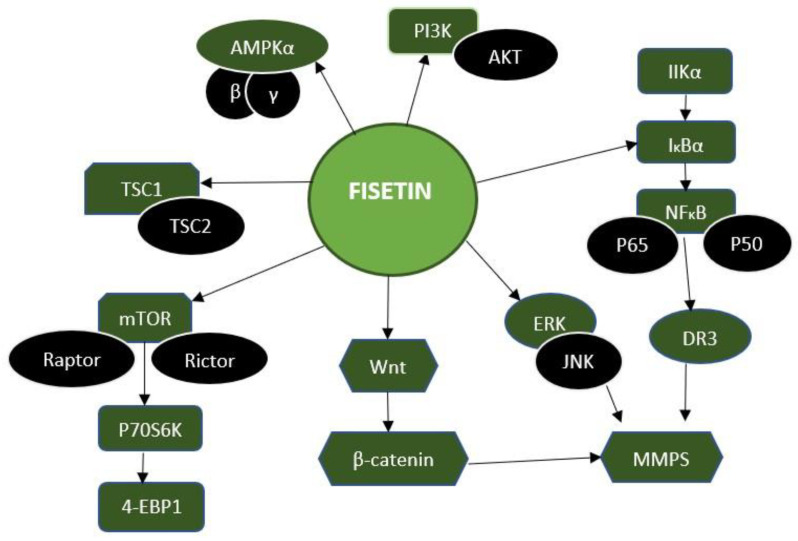
Molecular targets of fisetin. Fisetin inhibits the several signaling pathways, such as AMPK, PI3K, NF-κB, Wnt, mTOR, and TCS1, and increases related mRNA expressions such as p50, P65, and JNK, which are associated with promoting the apoptosis mechanism inside the cells. Hence, fisetin increased the death of cancer cells, and reduced their proliferation.

Fisetin rapidly compromises the proteasome-dependent microtubule drug-induced mitotic inhibition in numerous cell lines. As a result, chromosomal segregation begins early and, in unaffected tumour cells, leaves mitosis without typical cytokinesis. A cell culture study that looked at how fisetin affected the phosphorylation and localization of various mitotic proteins found that when it was introduced to the media, Cenp-F, Bub1, BubR1, and Aurora B soon lost their localization to the kinetochore and centromere. In addition, fisetin’s primary target was Aurora B kinase, whose activity is essentially decreased by fisetin in vitro [44]. Fisetin works on several cellular pathways, such as Wnt, Akt-PI3K, and ERK, as an inhibitor (Figure 4).

Lymphoblastoid cell lines were used to study the genotoxic effect of fisetin, and the commencement of micronuclei and segregating chromosomes was analyzed in the cells. Olaharski et al. used the CREST micronucleus assay to differentiate the micronuclei occurring due to chromosomal loss (CREST-positive) from those from chromosomal breakage (CREST-negative) in drug-treated cells. The rise in CREST-positive micronuclei indicated a lower concentration of fisetin, showing a genotoxic effect due to the loss of chromosomes [59]. Moreover, fisetin hinders the segregation of chromosomes by inhibiting the nuclear enzyme topoisomerase II-a, which is crucial for DNA replication. Hence, fisetin acts as an aneugen (affecting the mitotic spindle apparatus and cell division, which results in a gain or loss of chromosomes, leading to aneuploidy) and clastogen (creates fragmented chromosomes, causing a fragment of chromosome to be deleted/added/rearranged). Apoptotic cell death induced by fisetin was observed in various cancer cell lines. Reports have shown that the antiproliferative and proapoptotic effects mediated by fisetin specifically target cancer cells, leaving healthy cells unaffected. The selective effect of fisetin is attributed to the differential modulation of cell signaling pathways in the tumor mass compared to their nontumor counterparts [11].

## 4. Pharmacokinetics and Bioavailability of Fisetin

Fisetin exhibits a very short terminal half-life of approximately 3 hrs in its free form. This half-life is found to be less than that of its metabolites [60]. Fisetin and its metabolites were tested in rats, and their effects on hemolysis brought on by 2,2’-azobis (2-amidinopropane hydrochloride) (AAPH) were compared. The mean concentration–time profiles of metabolites in serum rapidly decreased with fisetin, at a dose of 10 mg/kg (intravenously). Higher concentrations of sulfates/glucuronides were present at all time points than the parent compounds, indicating liver-biotransformed fisetin by conjugation metabolism (sulfation). The level of fisetin was maintained on oral dosage (50 mg/kg weight of the body) after the first pass due to the existence of the parent component in serum. Fisetin was converted to sulphates and glucuronides, whereas enterocytes underwent sulfation less frequently than hepatocytes. Following treatment with 50 mg/kg of fisetin, the Cmax and AUC_0-4320min_ (area under serum concentration–time curve 0 to 4320 min) values of the 5-OH-flavone sulfate/glucuronide were 27 and 59 times greater, respectively, than those of the 5-OH-flavone after 40 milligrams/kg of the body weight of 5-OH-flavone. The AUC_0-4320 min_ of 7-OH-flavone sulfate/glucuronide was found to be significantly lower than that of 5-OH-flavone sulfate/glucuronide after an equivalent dosage. Fisetin and its serum metabolites prevented hemolysis brought on by AAPH, showing that the residual phenolic groups’ post-conjugation metabolism is responsible for their scavenging free-radical actions [61]. Following intraperitoneal delivery of the drug to mice at a dose of 223 mg/kg, the Cmax reached 2.5 µg/mL in 15 min. There was a biphasic decline in plasma concentration, with a short half-life of 0.09 h and a terminal half-life of 3.1 h.

The bioavailability of fisetin was enhanced by employing several formulation approaches, the majority of which are based on the application of nanotechnology. For instance, fisetin-loaded nanocochleates improved drug bioavailability up to 141 times following sustained release of the drug from the prepared complex [62]. Additionally, the drug solubility was also improved by 6.5-fold by complexation with cyclodextrin [63]. The fisetin-loaded liposomal system improved the drug bioavailability 47 times after intraperitoneal injection. At a dose of 21 mg/kg, liposomal fisetin inhibited tumor growth more than two-fold compared to pure drug alone [64]. Another approach to enhance the bioavailability and solubility of fisetin is to prepare crystalline nanosuspensions using Eudragit^®^EPO, stabilizers, surfactants, and polymers [65].

## 5. Novel Formulation Strategies and Drug Delivery System of Fisetin

**Figure 5:**
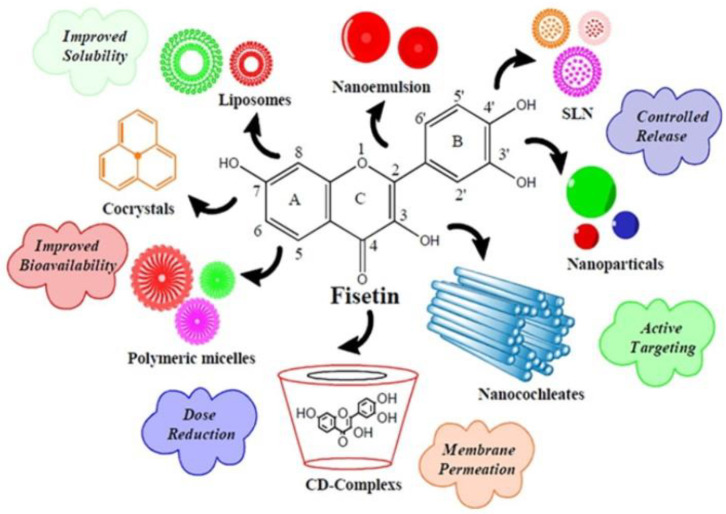
Delivery strategies used to improve fisetin performance. Adapted from [64]. Copyright © 2018 Elsevier Masson SAS.

Fisetin, being a phytopharmaceutical, has an advantage over synthetic drugs due to its safety profile and biocompatibility. Fisetin may be considered a prime candidate for use as an effective anticancer agent, due to its ability to affect various signaling pathways. Unfortunately, poor targeting and stability issues due to its undesirable hydrophobic nature and extremely poor aqueous solubility (<1 mg/mL) make it challenging to administer intravenously, leading to compromised bioavailability and limiting its use. To address this issue and overcome the hurdles related to drug delivery, it is crucial to develop novel delivery strategies to increase bioavailability and eventually increase the therapeutic outcome. Intensive research has been carried out to develop drug carriers for flavonoids. The use of biodegradable and biocompatible polymers in nanotechnology-based delivery systems helps overcome these challenges (Figure 5, Table 2).

### 5.1. Complexation

Complexation improves the physicochemical stability, dissolution rate, solubility, and bioavailability of poor water-soluble drugs [59]. Cyclodextrins are highly versatile oligosaccharides that are widely used as pharmaceutical excipients for this purpose. Cyclodextrin derivatives can help substances with poor water solubility become more soluble. Additionally, P-glycoprotein (P-GP), which is in charge of drug efflux, and cytochrome P450, which is in charge of drug metabolism and improves oral bioavailability, are inhibited by cyclodextrins [78]. It has been discovered that making a fisetin-hydroxyl propyl beta-cyclodextrin (HPbCD) inclusion complex (FHIC) increases fisetin’s solubility and, consequently, its bioavailability [79]. 

In another study, fisetin was complexed with three types of cyclodextrin to improve solubility. The researcher found better solubility when fisetin was complexed with sulfobutylethere-b-cyclodextrin. Furthermore, the addition of 20% v/v ethanolic solution enhanced the solubilization of fisetin by 5.9 times, compared to water alone [80]. Similarly, the complexation of the fisetin and cyclosophoraose dimer improved the drug solubility by 6.5 times. The solubility of dimer was 2.4 times more compared to its b-cyclodextrin complex. The dimer used for the complexation showed higher cytotoxicity of fisetin than pure fisetin in Hela cells [63]. 

### 5.2. Self-Nanoemulsifying Drug Delivery System (SNEDDS)

SNEDDS is an isotropic anhydrous mixture of oils, surfactants (HLB>12), and cosurfactants. This system not only improves the solubility and bioavailability of the active ingredient, but also provides better stability, processing control, and reproducibility. Moreover, it offers a lower production budget with enhanced patient compliance [80]. SNEDDS performs a dual action of increasing molecule solubility and providing protection to the gastrointestinal tract. In a study, the drug was incorporated into a nanoemulsion to enhance fisetin’s therapeutic and pharmacokinetic profile. No significant difference compared to free fisetin seemed to appear upon systemic exposure in mice after intravenous administration. However, upon intraperitoneal administration, fisetin exhibited 24 times higher bioavailability than free fisetin-treated mice at lower doses [70]. Similarly, the SNEDDS consisting of castor oil, lauroglycol, Tween 80, and transcutol were made to enhance the drug solubility of fisetin. The in vitro cell line results suggested that the fisetin-loaded SNEDDS had 3.79-fold higher cellular permeation than the free drug [69].

### 5.3. Lipid Vesicles

Liposomes have been found to be useful in increasing the accumulation of fisetin within tumors. A study conducted in vivo on mice revealed that the bioavailability of liposomal fisetin was 47 times greater than that of free fisetin [62]. Other vesicular carrier systems that have been explored for the delivery of fisetin are ethosomes and glycerosomes. Both of these are phospholipidic vesicles with high bilayer fluidity used for dermal and transdermal drug delivery [81,82]. Glycerosomes loaded with the drug fisetin displayed added benefits, such as enhanced penetration of the drug into deeper layers of the skin due to glycerin, resulting in lipid fluidization and hydration of the skin. Hence, it is primarily used for dermal applications of fisetin [65]. In vivo studies inferred that liposomes could remain stable for 59 days, retaining their antitumor activity in different tumors and endothelial cell lines [74]. Drug-loaded binary ethosomes were applied to the skin for the treatment of skin cancer. They showed sustained release behavior and improved penetration into the skin of rhodamine B-loaded endosome formulation, which was an added advantage. In vivo studies showed increased Cskin-max and AUC_0-8h_ (area under serum concentration–time curve 0 to 8 h) compared to conventional gel. It also showed a decrease in TNF-α and IL-α in mice pretreated with binary endosomes compared to mice exposed to UV only [72]. Furthermore, Mohapatra et al. (2011) investigated whether fisetin could be an effective fluorescent probe for lipid membranes. The fisetin was bound to the sensing lipid bilayer membrane and used as membrane expulsive target to enhance the antioxidant activity [49].

### 5.4. Lipid-Based Nanoparticles

Lipid-based nanocarriers, such as solid lipid nanoparticles (SLNs), nanostructured lipid carriers (NLCs), and nano-emulsions, are being utilized for the delivery and targeting of highly lipophilic drugs, including fisetin [83]. Kulbacka et al. (2016) reported in their study that they prepared multifunctional SLNs loaded with cyanine-type IR-780 as a photosensitizer/diagnostic agent, along with fisetin or baicalein, to explore the potential of combination therapy in cancer eradication. They stated that delivery of drugs through these carriers was precise and depicted tumor growth inhibition with lower toxicity [84]. The glycerol monostearate-, sodium deoxycholate- and sodium cholate-based SLNs loaded with fisetin improved the photophysical properties of the drug, and were photostable at room temperature. The fisetin did not show any polymorphic transformation during storage when loaded in a lipid-based nanocarrier [85]. Similarly, the fisetin-loaded nano-emulsion was prepared using miglyol 812 N, lipoid E 80, labrasol, Tween 80, and water. No significant changes were observed in the pharmacokinetic profile of the fisetin-loaded nano-emulsion after IV injection (13 mg/kg) compared to that of pure drug. However, intraperitoneal administration improved the bioavailability of the drug by 24-fold. The antitumor activity of the fisetin-loaded nanoemulsion was shown at a concentration of 36.6 mg/kg, which was far lower than that of free drug (223 mg/kg), against a Lewis lung carcinoma model in mice. The results demonstrated that solubilization of fisetin was improved by the nano-emulsion, and it depicted enhanced antitumor activity [70].

### 5.5. Polymeric Micelles and Nanoparticles

Polymeric micelles are formed by amphiphilic block copolymers, which possess nanoscopic core/shell structures [86]. These systems are used to entrap drugs, resulting in increased anticancer activity against ovarian carcinoma by destroying the tumor mass and inhibiting further multiplication of cells [87]. Fisetin-loaded albumin nanoparticles were prepared using the desolvation method. It displayed advantages such as improved bioavailability, good entrapment, and delivery to a specific target site [75].

In another study, fisetin-loaded polymeric micelles composed of TPGS-PLA exhibited dose-dependent cytotoxicity against MCF-7 cells. The delivery of fisetin through polymeric micelles enhanced the cytotoxic effect in breast cancer cell lines, and induced 42% cell apoptosis at 48 h compared with free fisetin, which showed only 30% cell apoptosis at a similar time. Moreover, it reduced the tumor burden in mice, induced cell apoptosis, and reduced the tumor mass (which tumor) [77]. 

Similarly, monomethyl poly(ethylene glycol)-poly(ε-caprolactone) polymeric micelles loaded with fisetin were evaluated for anticancer efficacy against ovarian cancer, and showed induced cell apoptosis in a dose-dependent manner in SKOV3 cells. The fisetin-loaded micelles exhibited reduced tumor growth, enhanced tumor apoptosis, and angiogenesis inhibition [71].

The delivery of fisetin also shows promising results with polymeric nanoparticles. The poly(lactic acid) nanoparticle (PLA-NP)-based fisetin formulation was found to enhance fisetin solubility and therapeutic efficacy against HCT116 colon cancer cells in vitro and xenograft 4T1 breast cancer in vivo [66]. Similarly, human serum albumin-based nanoparticles (HSA-NPs) were also developed, with an aim to improve the bioavailability of fisetin. The potent antioxidant effects of fisetin-loaded HSA-NPs were confirmed by the DPPH assay, and the results demonstrated the capabilities associated with the developed system, to deliver fisetin efficiently [67]. 

Furthermore, polymeric nanoparticles made by poly-(ε-caprolactone) (PCL) and PLGA-PEG-COOH that were loaded with fisetin depicted the controlled release of the drug in simulated gastric as well as intestinal conditions. The nanocarriers were prepared with the aim of delivering fisetin for antioxidant as well as antihyperglycemic effects, and to observe the stability of encapsulated fisetin. The process for developing nanoparticles was efficient enough to retain the DPPH and ABTS scavenging capacity, as well as α-glucosidase inhibition activity [26]. In another study, poly(vinyl pyrrolidone) polymeric nanoparticles (PVP-NPs) processed through a supercritical antisolvent (SAS) method improved the hydrophilicity of fisetin. As a result, the anticancer efficacy, pharmacokinetics, and bioavailability of the fisetin was improved [88].6. Toxicity and Clinical Status of Fisetin

Potential chemotherapeutic drugs kill cancerous as well as healthy cells, and demonstrate undesired side effects. To minimize the side effects and improve the therapeutic outcomes of cancer therapy, plant-based nutritional supplements are currently being explored. Flavonoids offer great potential to eliminate cancer cells and provide protection to healthy cells via antioxidant properties. Recently, fisetin has been used against several types of cancers, and exhibited much fewer side effects than other chemotherapeutic agents [89]. Fisetin is an ingredient available in common plant-based foods, and has reported no adverse effects. Irrespective of the benefits bestowed by fisetin to treat breast cancer, thorough scrutiny of its toxicity is needed, as it requires a high dose to offer therapeutic benefits. Despite numerous scientific interventions performed on animals, no severe toxicological evidence has been observed, even at higher drug levels. 

Fisetin-related clinical trials are limited in cancer therapy. As per the NIH-clinical trials database, only two studies are listed. In one of the phase 2 studies, the researchers are investigating the effect of fisetin to improve the physical function in postmenopausal women after receiving chemotherapy for stage I–III breast cancer [90]. In another study, the efficacy, safety and tolerability of the two different senolytic therapies, which include fisetin, and dasatinib plus quercetin, are being investigated in adult survivors of childhood cancer under phase 2 [91]. However, the level of safety should also be evaluated by conducting clinical trials. The major disadvantage of fisetin is that its aqueous solubility could be addressed by converting the drug polymer complex system by synthesizing a nanocarrier system. Several studies have shown that the drug’s solubility, bioavailability and dose, along with its therapeutic efficacy, were improved without any side effects. One such clinical trial on cancer patients reported that the fisetin treatment group experienced stomach discomfort [13]. It has also been observed that fisetin lowers the blood glucose level in diabetic animals, which implies a further reduction in blood glucose levels when co-administered with antihyperglycemic drugs [92,93]. Additionally, metabolism in the liver, as both warfarin and fisetin are processed in the same way, may result in an increased effect of warfarin over time [94]. 

## 6. Patents Related to Fisetin

Fisetin patents have primarily described its various preparation techniques and effective treatments. Primarily reported methods of fisetin preparation are based on extraction from microbial sources, and conversion of fisetin from fustin [95]. Several patents have exemplified its application in the treatment of prostate cancer, senile dementia, uterine myoma, acute pancreatitis, depression, neurodegenerative diseases, gastritis and gastric ulcer, and as an antihypertensive [96]. Additionally, fisetin application for skin disorders, such as skin regeneration, anti-aging effects, prevention of hair loss, and stimulation of hair growth, have also been reported. Few other studies were related to its role as an antioxidant, antimicrobial, weight loss agent, and memory enhancer (Table 3).

## 7. Conclusion and Future Prospects

Fisetin is a naturally occurring polyphenol that is considered to possess pleiotropic pharmacological properties, making it a potential candidate in the treatment of cancer and a few other diseases mentioned earlier. The major drawback is that its hydrophobic nature restricts its clinical use, due to its undesirable bioavailability profile. To overcome this hurdle, various formulation-based strategies, such as micelles, liposomes, nanoparticles, nanocochleates, SNEDDSs, and SLNs, have been used to improve its solubility and enhance its therapeutic effect. Future aspects of the fisetin delivery system include self-assembled lipid vesicles such as niosomes, ethosomes, cubosomes, and hexosomes. Research related to macromolecules and ligand-conjugated delivery, such as dendrimers, can be explored. These delivery strategies have the potential to reach clinics in the future. Future research needs to focus on augmenting the existing formulation flaws.

## Figures and Tables

**Table 1:** Physicochemical properties of fisetin.

Characteristics	Description
Occurrence	Cucumber, Apple, Strawberry, Grape, Persimmon, Onion
Chemical class	Flavonoid
IUPAC	2-(3,4-dihydroxyphenyl)-3,7-dihydroxychromen-4-one
Chemical formula	C_15_H_10_O_6_
Molecular mass	286.24 g/mol
Melting point	330.0 °C
Density	1.688 g/mL
Solubility	Soluble in ethanol, acetone, acetic acid, DMSO, solutions of fixed alkali hydroxides, methanol
BCS class	Class II (Low soluble and highly permeable)
Appearance	Crystalline powder
Log P	3.2
pKa	7.42
Chemical metabolites	Geraldol (3,4′,7-trihydroxy-3′-methoxyflavone), Fisetin-4′-glucoside
Stability	Stability ≥ 4 years at −20 °C

**Table 2:** Fisetin-based formulations developed using novel strategies.

Formulation Type	Preparation Method	Components	Indication	Findings	Comments	References
Soft nanovesicles (glycerol-based)		Glycerosomes for dermal delivery of fisetin	Skin cancer	Enhanced penetration into skin	It was suitable for dermal application	[65]
Nanocochleates	Trapping method	Fisetin, DMPC, cholesterol, ethanol, calcium chloride	Breast cancer	Improved therapeutic efficacy	It enhanced anticancer action, safety and bioavailability	[59]
Poly (lactic acid) nanoparticles	Spontaneous emulsification solvent diffusion (SESD) method	Poly-D,L-lactide, fisetin, Poloxamer 188 and acetonitrile	Colon cancer, breast cancer	Enhance drug solubility and therapeutic action	It enhanced anticancer action	[66]
Albumin-based nanoparticles	Desolvation method	Albumin and glutaraldehyde	Breast cancer	Increased solubility and stability	It enhanced the action against breast cancer cells	[67]
Inclusion complex in polymeric nanoparticles	Encapsulation into PLGA NPs as HPbCD complex	Hyroxypropyl b-cyclodextrin	Breast cancer	Higher drug loading capacity, enhanced bioavailability and anticancer action	Improved bioavailability and pharmacokinetics properties	[68]
Self-nano-emulsifying system		Lauroglycol FCC, Transcutol P, tween 80, Castor oil	Cancer, Parkinson’s disease	Improved biopharmaceutical properties like dissolution and rate of permeability	It was nontoxic and showed stability on change in temperature, dilution and pH	[69]
Nano-emulsion formulation		Miglyol 812N, Tween 80, water, Labrasol, Lipoid E80	Antitumor (Lung carcinoma)	Increase in bioavailability by 24-fold	Improved bioavailability and antitumor action	[70]
Nano-encapsulation	Nanoprecipitation method	(PCL) and PLGA-PEG-COOH	Antioxidant activity and anti-hyperglycemic effect	FS release is protected and preserved in gastric simulated conditions, and controls intestinal release	It controlled the release of antioxidant and anti-hyperglycemic FS	[25]
Polymeric micelles	The micelles self-assemble into structures.	Monomethyl poly, ε-caprolactone	Ovarian cancer	Enhanced cytotoxicity and apoptosis induction	Enhanced solubility and bioavailability	[71]
Binary ethosomes		Fisetin, Phospholipid 90G. Ethanol, Chloroform, sodium hydroxide	Management of skin cancer	Improved dermal delivery of the fisetin	Primarily used in management of skin cancer	[72]
Spherulites	Shearing of a lipidic lamellar phase is subjected to dispersion	Polyoxyethylene sorbitan ester, LipoidÒ E80, Polysorbate 80	Anticancer	Encapsulation potential is higher and slow-release capacity	Increased encapsulated payload of a hydrophobic compound	[73]
Liposomal formulation		DMSO, Cholesterol, phospholipids, Hepes/phosphate buffers	Anticancer	It had cytotoxicity and morphological effect	It is suitable for in vivo administration	[74]
Liposomal encapsulation		Formulation developed using DOPC and DODA-PEG2000	Antitumor (Lung carcinoma)	Increased bioavailability upon 47-fold	Enhanced bioavailability and anticancer effect	[62]
Bacterial cellulose scaffold	Prepared from Gluconaceter xylinus		Treating bone defects	No toxic effect over increased cell viability	Induced osteogenic differentiation and localized delivery	[75]
Folate functionalized pluronic micelles	Thin-film hydration method	Fisetin, Pluronic F127 (PF), Folic Acid, Di-cyclohexyl carbodiimide, Carbonyl di-imidazole	Breast cancer targeting	It increases solubility, bioavailability and active targetability increased its therapeutic efficacy	Six-fold increase in bioavailability and prolonged circulation time, plasma elimination and no toxicity	[76]
α-Tocopherol-Poly (lactic acid)-Based Polymeric Micelles		L,L-lactide, D-α-tocopheryl polyethylene glycol 1000 succinate	Breast Cancer	Higher cellular uptake	Effective in treatment of breast cancers	[77]

**Table 3:** List of patents filed on fisetin formulations.

Title	Applications	Description	References
Application of fisetin in inhibiting proliferation of pancreatic cancer cells and mouse pancreatic cancer tumors	Inhibition of pancreatic cancer tumor proliferation	The invention provides new uses of fisetin to inhibit pancreatic cancer cell and mouse pancreatic cancer tumor proliferation to reduce the deficiencies of existing pancreatic cancer treatment methods.	[97]
Method for preparing Rhus verniciflua Stokes extract containing increased fisetin content, and metastasis-inhibiting anticancer agent composition containing same	Improvement in anti-cancer property of fisetin	The invention includes a method for preparing a Rhus verniciflua Stokes extract containing increased fisetin content by converting fustin to fisetin by adding an extract concentrate and reacting at least one catalyst consisting of platinum, chromium, nickel, silicon, copper, and oxides of said metals; and forming a cancer-preventing composition.	[98]
Method of administering fisetin through oral, transdermal or topical dosage form	Treatment of androgen-dependent prostate cancer in males.	Fisetin treatment inhibits PI3K and Akt, resulting in inhibition of cell growth followed by apoptosis of human prostate cancer LNCaP cells.	[99]
Application of fisetin in combined gemcitabine pancreatic cancer treatment	Combination treatment for pancreatic cancer	The invention provides an application of fisetin in the treatment of pancreatic cancer in combination with gemcitabine; that is, its use increases the curative effect of gemcitabine chemotherapy, thereby making up for the deficiency in existing pancreatic cancer chemotherapy drug resistance.	[100]
4’-substituted analogues of fisetin and their use in the treatment of cancer	For the treatment of cancer	This invention relates to novel compounds that are 4’-substituted analogues of the flavonol fisetin. The invention further provides for pharmaceutical compositions comprising these compounds, and the use of these compounds and compositions in the treatment of cancer in particular, but not exclusively, in the treatment of epithelial cancers.	[101]
Methods of treating brain cancer and related diagnostic methods	For the treatment of medulloblastoma	Treating medulloblastoma using a combination of STAT3 and YB-1 inhibitor, that is, 2-(3,4-dihydroxyphenyl)-3,7-dihydroxy-4H-chromen-4-one (fisetin), and also for diagnostic purposes.	[102]
Composition comprising phenolic compound for prevention and treatment of liver cancer	For the treatment of liver cancer	The invention discovered a compound that inhibits the proliferation of liver cancer cells in some phenolic compounds, and a composition containing the same has the effect of preventing and treating liver cancer.	[103]

## Data Availability

Data sharing not applicable.

## References

[1] World Health Organization (2020). Latest Global Cancer Data: Cancer Burden Rises to 19.3 Million New Cases and 10.0 Million Cancer Deaths in 2020.

[2] Sung H., Ferlay J., Siegel R.L., Laversanne M., Soerjomataram I., Jemal A., Bray F. (2021). Global Cancer Statistics 2020: GLOBOCAN Estimates of Incidence and Mortality Worldwide for 36 Cancers in 185 Countries. CA Cancer J. Clin..

[3] Liberti M.V., Locasale J.W. (2016). The Warburg Effect: How Does It Benefit Cancer Cells?. Trends Biochem. Sci..

[4] Cao Y. (2017). Tumorigenesis as a Process of Gradual Loss of Original Cell Identity and Gain of Properties of Neural Precursor/Progenitor Cells. Cell Biosci..

[5] Lindsey S., Langhans S.A. (2014). Crosstalk of Oncogenic Signaling Pathways during Epithelial-Mesenchymal Transition. Front. Oncol..

[6] Kontomanolis E.N., Koutras A., Syllaios A., Schizas D., Mastoraki A., Garmpis N., Diakosavvas M., Angelou K., Tsatsaris G., Pagkalos A. (2020). Role of Oncogenes and Tumor-Suppressor Genes in Carcinogenesis: A Review. Anticancer Res..

[7] Korgaonkar N., Yadav K.S. (2019). Understanding the Biology and Advent of Physics of Cancer with Perspicacity in Current Treatment Therapy. Life Sci..

[8] Sak K. (2014). Cytotoxicity of Dietary Flavonoids on Different Human Cancer Types. Pharmacogn. Rev..

[9] Raffa D., Maggio B., Raimondi M.V., Plescia F., Daidone G. (2017). Recent Discoveries of Anticancer Flavonoids. Eur. J. Med. Chem..

[10] Hodek P., Trefil P., Stiborová M. (2002). Flavonoids-Potent and Versatile Biologically Active Compounds Interacting with Cytochromes P450. Chem. Biol. Interact..

[11] Jang H.S., Kook S.H., Son Y.O., Kim J.G., Jeon Y.M., Jang Y.S., Choi K.C., Kim J., Han S.K., Lee K.Y. (2005). Flavonoids Purified from Rhus Verniciflua Stokes Actively Inhibit Cell Growth and Induce Apoptosis in Human Osteosarcoma Cells. Biochim. Biophys. Acta-Gen. Subj..

[12] Moon Y.J., Wang X., Morris M.E. (2006). Dietary Flavonoids: Effects on Xenobiotic and Carcinogen Metabolism. Toxicol. In Vitro.

[13] Sun X., Ma X., Li Q., Yang Y., Xu X., Sun J., Yu M., Cao K., Yang L., Yang G. (2018). Anti-cancer Effects of Fisetin on Mammary Carcinoma Cells via Regulation of the PI3K/Akt/MTOR Pathway: In Vitro and in Vivo Studies. Int. J. Mol. Med..

[14] Zhu J., Zhang H., Zhu Z., Zhang Q., Ma X., Cui Z., Yao T. (2015). Effects and Mechanism of Flavonoids from Astragalus Complanatus on Breast Cancer Growth. Naunyn. Schmiedebergs. Arch. Pharmacol..

[15] Dinakar Y.H., Kumar H., Mudavath S.L., Jain R., Ajmeer R., Jain V. (2022). Role of STAT3 in the Initiation, Progression, Proliferation and Metastasis of Breast Cancer and Strategies to Deliver JAK and STAT3 Inhibitors. Life Sci..

[16] Nde C., Zingue S., Winter E., Creczynski-Pasa T., Michel T., Fernandez X., Njamen D., Clyne C. (2015). Flavonoids, Breast Cancer Chemopreventive and/or Chemotherapeutic Agents. Curr. Med. Chem..

[17] Abotaleb M., Samuel S.M., Varghese E., Varghese S., Kubatka P., Liskova A., Büsselberg D. (2019). Flavonoids in Cancer and Apoptosis. Cancers.

[18] Syed D., Adhami V., Khan M., Mukhtar H. (2013). Inhibition of Akt/MTOR Signaling by the Dietary Flavonoid Fisetin. Anticancer. Agents Med. Chem..

[19] Ravichandran N., Suresh G., Ramesh B., Vijaiyan Siva G. (2011). Fisetin, a Novel Flavonol Attenuates Benzo(a)Pyrene-Induced Lung Carcinogenesis in Swiss Albino Mice. Food Chem. Toxicol..

[20] Touil Y.S., Seguin J., Scherman D., Chabot G.G. (2011). Improved Antiangiogenic and Antitumour Activity of the Combination of the Natural Flavonoid Fisetin and Cyclophosphamide in Lewis Lung Carcinoma-Bearing Mice. Cancer Chemother. Pharmacol..

[21] Syed D.N., Afaq F., Maddodi N., Johnson J.J., Sarfaraz S., Ahmad A., Setaluri V., Mukhtar H. (2011). Inhibition of Human Melanoma Cell Growth by the Dietary Flavonoid Fisetin Is Associated with Disruption of Wnt/β-Catenin Signaling and Decreased Mitf Levels. J. Investig. Dermatol..

[22] Murtaza I., Adhami V.M., Hafeez B.B., Saleem M., Mukhtar H. (2009). Fisetin, a Natural Flavonoid, Targets Chemoresistant Human Pancreatic Cancer AsPC-1 Cells through DR3-Mediated Inhibition of NF-ΚB. Int. J. Cancer.

[23] Bhat T.A., Nambiar D., Pal A., Agarwal R., Singh R.P. (2012). Fisetin Inhibits Various Attributes of Angiogenesis in Vitro and in Vivo-Implications for Angioprevention. Carcinogenesis.

[24] Mohd Yusof Y.A. (2016). Gingerol and Its Role in Chronic Diseases. Adv. Exp. Med. Biol..

[25] Rengarajan T., Yaacob N.S. (2016). The Flavonoid Fisetin as an Anticancer Agent Targeting the Growth Signaling Pathways. Eur. J. Pharmacol..

[26] Sechi M., Syed D.N., Pala N., Mariani A., Marceddu S., Brunetti A., Mukhtar H., Sanna V. (2016). Nanoencapsulation of Dietary Flavonoid Fisetin: Formulation and in Vitro Antioxidant and α-Glucosidase Inhibition Activities. Mater. Sci. Eng. C.

[27] Li J., Cheng Y., Qu W., Sun Y., Wang Z., Wang H., Tian B. (2011). Fisetin, a Dietary Flavonoid, Induces Cell Cycle Arrest and Apoptosis through Activation of P53 and Inhibition of NF-Kappa B Pathways in Bladder Cancer Cells. Basic Clin. Pharmacol. Toxicol..

[28] PeiMing Y., HoHsing T., ChihWen P., WenShu C., ShuJun C. (2012). Dietary Flavonoid Fisetin Targets Caspase-3-Deficient Human Breast Cancer MCF-7 Cells by Induction of Caspase-7-Associated Apoptosis and Inhibition of Autophagy. Int. J. Oncol..

[29] Kang K.A., Piao M.J., Hyun J.W. (2015). Fisetin Induces Apoptosis in Human Nonsmall Lung Cancer Cells via a Mitochondria-Mediated Pathway. Vitr. Cell. Dev. Biol.-Anim..

[30] Drug Bank. https://go.drugbank.com/Drugs/DB07795..

[31] Kashyap D., Sharma A., Sak K., Tuli H.S., Buttar H.S., Bishayee A. (2018). Fisetin: A Bioactive Phytochemical with Potential for Cancer Prevention and Pharmacotherapy. Life Sci..

[32] Fisetin Item No. 15246. https://www.caymanchem.com/pdfs/15246.pdf..

[33] Kimira M., Arai Y., Shimoi K., Watanabe S. (1998). Japanese Intake of Flavonoids and Isoflavonoids from Foods. J. Epidemiol..

[34] Surnis S.A., Patil P.S., Jadhav R.H. (2016). Extraction, Isolation and Quantification of Bioactive Compound (Fisetin) and Its Product Formulation. Int. J. Eng. Res..

[35] Tsurudome N., Minami Y., Kajiya K. (2022). Fisetin, a Major Component Derived from Mulberry (Morus Australis Poir.) Leaves, Prevents Vascular Abnormal Contraction. BioFactors.

[36] Direito R., Reis C., Roque L., Gonçalves M., Sanches-Silva A., Gaspar M.M., Pinto R., Rocha J., Sepodes B., Bronze M.R. (2019). Phytosomes with Persimmon (Diospyros Kaki l.) Extract: Preparation and Preliminary Demonstration of in Vivo Tolerability. Pharmaceutics.

[37] Kostanecki S., Lampe V., Tambor J. (1904). Synthese Des Fisetins. Ber. der Dtsch. Chem. Ges..

[38] Grynkiewicz G., Demchuk O.M. (2019). New Perspectives for Fisetin. Front. Chem..

[39] Lu H., Chang D.J., Baratte B., Meijer L., Schulze-Gahmen U. (2005). Crystal Structure of a Human Cyclin-Dependent Kinase 6 Complex with a Flavonol Inhibitor, Fisetin. J. Med. Chem..

[40] Kim J.A., Lee S., Kim D.E., Kim M., Kwon B.M., Han D.C. (2015). Fisetin, a Dietary Flavonoid, Induces Apoptosis of Cancer Cells by Inhibiting HSF1 Activity through Blocking Its Binding to the Hsp70 Promoter. Carcinogenesis.

[41] Kammerud S.C., Metge B.J., Elhamamsy A.R., Weeks S.E., Alsheikh H.A., Mattheyses A.L., Shevde L.A., Samant R.S. (2021). Novel Role of the Dietary Flavonoid Fisetin in Suppressing RRNA Biogenesis. Lab Investig..

[42] Salmela A.L., Pouwels J., Varis A., Kukkonen A.M., Toivonen P., Halonen P.K., Perälä M., Kallioniemi O., Gorbsky G.J., Kallio M.J. (2009). Dietary Flavonoid Fisetin Induces a Forced Exit from Mitosis by Targeting the Mitotic Spindle Checkpoint. Carcinogenesis.

[43] Sung B., Pandey M.K., Aggarwal B.B. (2007). Fisetin, an Inhibitor of Cyclin-Dependent Kinase 6, down-Regulates Nuclear Factor-ΚB-Regulated Cell Proliferation, Antiapoptotic and Metastatic Gene Products through the Suppression of TAK-1 and Receptor-Interacting Protein-Regulated IκBα Kinase Activatio. Mol. Pharmacol..

[44] Adhami V.M., Syed D.N., Khan N., Mukhtar H. (2012). Dietary Flavonoid Fisetin: A Novel Dual Inhibitor of PI3K/Akt and MTOR for Prostate Cancer Management. Biochem. Pharmacol..

[45] Pal H.C., Sharma S., Strickland L.R., Katiyar S.K., Ballestas M.E., Athar M., Elmets C.A., Afaq F. (2014). Fisetin Inhibits Human Melanoma Cell Invasion through Promotion of Mesenchymal to Epithelial Transition and by Targeting MAPK and NFκB Signaling Pathways. PLoS ONE.

[46] Noh E.M., Park Y.J., Kim J.M., Kim M.S., Kim H.R., Song H.K., Hong O.Y., So H.S., Yang S.H., Kim J.S. (2015). Fisetin Regulates TPA-Induced Breast Cell Invasion by Suppressing Matrix Metalloproteinase-9 Activation via the PKC/ROS/MAPK Pathways. Eur. J. Pharmacol..

[47] Smith M.L., Murphy K., Doucette C.D., Greenshields A.L., Hoskin D.W. (2016). The Dietary Flavonoid Fisetin Causes Cell Cycle Arrest, Caspase-Dependent Apoptosis, and Enhanced Cytotoxicity of Chemotherapeutic Drugs in Triple-Negative Breast Cancer Cells. J. Cell. Biochem..

[48] Imran M., Saeed F., Gilani S.A., Shariati M.A., Imran A., Afzaal M., Atif M., Tufail T., Anjum F.M. (2021). Fisetin: An Anticancer Perspective. Food Sci. Nutr..

[49] Mohapatra M., Mishra A.K. (2011). Photophysical Behavior of Fisetin in Dimyristoylphosphatidylcholine Liposome Membrane. J. Phys. Chem. B.

[50] Kim C.W., Yoon Y., Kim M.Y., Baik S.K., Ryu H., Park I.H., Eom Y.W. (2022). 12-O-Tetradecanoylphorbol-13-Acetate Reduces Activation of Hepatic Stellate Cells by Inhibiting the Hippo Pathway Transcriptional Coactivator YAP. Cells.

[51] Chuang J.Y., Chang P.C., Shen Y.C., Lin C., Tsai C.F., Chen J.H., Yeh W.L., Wu L.H., Lin H.Y., Liu Y.S. (2014). Regulatory Effects of Fisetin on Microglial Activation. Molecules.

[52] Chiruta C., Schubert D., Dargusch R., Maher P. (2012). Chemical Modification of the Multitarget Neuroprotective Compound Fisetin. J. Med. Chem..

[53] Youns M., Hegazy W.A.H. (2017). The Natural Flavonoid Fisetin Inhibits Cellular Proliferation of Hepatic, Colorectal, and Pancreatic Cancer Cells through Modulation of Multiple Signaling Pathways. PLoS ONE.

[54] Jankun J., Selman S.H., Aniola J., Skrzypczak-Jankun E. (2006). Nutraceutical Inhibitors of Urokinase: Potential Applications in Prostate Cancer Prevention and Treatment. Oncol. Rep..

[55] Chou R.H., Hsieh S.C., Yu Y.L., Huang M.H., Huang Y.C., Hsieh Y.H. (2013). Fisetin Inhibits Migration and Invasion of Human Cervical Cancer Cells by Down-Regulating Urokinase Plasminogen Activator Expression through Suppressing the P38 MAPK-Dependent NF-ΚB Signaling Pathway. PLoS ONE.

[56] Lu W., Zhu J., Zou S., Li X., Huang J. (2013). The Efficient Expression of Human Fibroblast Collagenase in Escherichia Coli and the Discovery of Flavonoid Inhibitors. J. Enzyme Inhib. Med. Chem..

[57] Olaharski A.J., Mondrala S.T., Eastmond D.A. (2005). Chromosomal Malsegregation and Micronucleus Induction in Vitro by the DNA Topoisomerase II Inhibitor Fisetin. Mutat. Res.-Genet. Toxicol. Environ. Mutagen.

[58] Shia C.-S., Tsai S.-Y., Kuo S.-C., Hou Y.-C., Chao P.-D.L. (2009). Metabolism and Pharmacokinetics of Antihemolysis Effects of Fisetin and Its Serum Metabolites. J. Agric. Food Chem..

[59] Bothiraja C., Yojana B.D., Pawar A.P., Shaikh K.S., Thorat U.H. (2014). Fisetin-Loaded Nanocochleates: Formulation, Characterisation, in Vitro Anticancer Testing, Bioavailability and Biodistribution Study. Expert Opin. Drug Deliv..

[60] Khan N., Syed D.N., Ahmad N., Mukhtar H. (2013). Fisetin: A Dietary Antioxidant for Health Promotion. Antioxidants Redox Signal..

[61] Jeong D., Choi J.M., Choi Y., Jeong K., Cho E., Jung S. (2013). Complexation of Fisetin with Novel Cyclosophoroase Dimer to Improve Solubility and Bioavailability. Carbohydr. Polym..

[62] Seguin J., Brullé L., Boyer R., Lu Y.M., Ramos Romano M., Touil Y.S., Scherman D., Bessodes M., Mignet N., Chabot G.G. (2013). Liposomal Encapsulation of the Natural Flavonoid Fisetin Improves Bioavailability and Antitumor Efficacy. Int. J. Pharm..

[63] Dzakwan M., Ganet E.P., Rachmat M., Wikarsa S. (2019). Nanosized and Enhancement of Solubility Fisetin. Asian J. Pharm. Res. Dev..

[64] Mehta P., Pawar A., Mahadik K., Bothiraja C. (2018). Emerging Novel Drug Delivery Strategies for Bioactive Flavonol Fisetin in Biomedicine. Biomed. Pharmacother..

[65] Moolakkadath T., Aqil M., Ahad A., Imam S.S., Praveen A., Sultana Y., Mujeeb M. (2020). Preparation and Optimization of Fisetin Loaded Glycerol Based Soft Nanovesicles by Box-Behnken Design. Int. J. Pharm..

[66] Feng C., Yuan X., Chu K., Zhang H., Ji W., Rui M. (2019). Preparation and Optimization of Poly (Lactic Acid) Nanoparticles Loaded with Fisetin to Improve Anti-Cancer Therapy. Int. J. Biol. Macromol..

[67] Ghosh P., Singha Roy A., Chaudhury S., Jana S.K., Chaudhury K., Dasgupta S. (2016). Preparation of Albumin Based Nanoparticles for Delivery of Fisetin and Evaluation of Its Cytotoxic Activity. Int. J. Biol. Macromol..

[68] Kadari A., Gudem S., Kulhari H., Bhandi M.M., Borkar R.M., Kolapalli V.R.M., Sistla R. (2017). Enhanced Oral Bioavailability and Anticancer Efficacy of Fisetin by Encapsulating as Inclusion Complex with HPβCD in Polymeric Nanoparticles. Drug Deliv..

[69] Kumar R.R., Khursheed R., Kumar R.R., Awasthi A., Sharma N., Khurana S., Kapoor B., Khurana N., Singh S.K., Gowthamarajan K. (2019). Self-Nanoemulsifying Drug Delivery System of Fisetin: Formulation, Optimization, Characterization and Cytotoxicity Assessment. J. Drug Deliv. Sci. Technol..

[70] Ragelle H., Crauste-Manciet S., Seguin J., Brossard D., Scherman D., Arnaud P., Chabot G.G. (2012). Nanoemulsion Formulation of Fisetin Improves Bioavailability and Antitumour Activity in Mice. Int. J. Pharm..

[71] Xiao X., Zou J., Fang Y., Meng Y., Xiao C., Fu J., Liu S., Bai P., Yao Y. (2018). Fisetin and Polymeric Micelles Encapsulating Fisetin Exhibit Potent Cytotoxic Effects towards Ovarian Cancer Cells. BMC Complement. Altern. Med..

[72] Moolakkadath T., Aqil M., Ahad A., Imam S.S., Praveen A., Sultana Y., Mujeeb M., Iqbal Z. (2019). Fisetin Loaded Binary Ethosomes for Management of Skin Cancer by Dermal Application on UV Exposed Mice. Int. J. Pharm..

[73] Crauste-Manciet S., Larquet E., Khawand K., Bessodes M., Chabot G.G., Brossard D., Mignet N. (2013). Lipidic Spherulites: Formulation Optimisation by Paired Optical and Cryoelectron Microscopy. Eur. J. Pharm. Biopharm..

[74] Mignet N., Seguin J., Romano M.R., Brullé L., Touil Y.S., Scherman D., Bessodes M., Chabot G.G. (2012). Development of a Liposomal Formulation of the Natural Flavonoid Fisetin. Int. J. Pharm..

[75] Vadaye Kheiry E., Parivar K., Baharara J., Fazly Bazzaz B.S., Iranbakhsh A. (2018). The Osteogenesis of Bacterial Cellulose Scaffold Loaded with Fisetin. Iran. J. Basic Med. Sci..

[76] Pawar A., Singh S., Rajalakshmi S., Shaikh K., Bothiraja C. (2018). Development of Fisetin-Loaded Folate Functionalized Pluronic Micelles for Breast Cancer Targeting. Artif. Cells Nanomed. Biotechnol..

[77] Wang L., Zhang D.Z., Wang Y.X. (2017). Bioflavonoid Fisetin Loaded α-Tocopherol-Poly(Lactic Acid)-Based Polymeric Micelles for Enhanced Anticancer Efficacy in Breast Cancers. Pharm. Res..

[78] Haimhoffer Á., Rusznyák Á., Réti-Nagy K., Vasvári G., Váradi J., Vecsernyés M., Bácskay I., Fehér P., Ujhelyi Z., Fenyvesi F. (2019). Cyclodextrins in Drug Delivery Systems and Their Effects on Biological Barriers. Sci. Pharm..

[79] Zhang J.Q., Jiang K.M., An K., Ren S.H., Xie X.G., Jin Y., Lin J. (2015). Novel Water-Soluble Fisetin/Cyclodextrins Inclusion Complexes: Preparation, Characterization, Molecular Docking and Bioavailability. Carbohydr. Res..

[80] Kazi M., Al-Swairi M., Ahmad A., Raish M., Alanazi F.K., Badran M.M., Khan A.A., Alanazi A.M., Hussain M.D. (2019). Evaluation of Self-Nanoemulsifying Drug Delivery Systems (SNEDDS) for Poorly Water-Soluble Talinolol: Preparation, in Vitro and in Vivo Assessment. Front. Pharmacol..

[81] Verma P., Pathak K. (2010). Therapeutic and Cosmeceutical Potential of Ethosomes: An Overview. J. Adv. Pharm. Technol. Res..

[82] Gupta P., Mazumder R., Padhi S. (2020). Glycerosomes: Advanced Liposomal Drug Delivery System. Indian J. Pharm. Sci..

[83] Jain V., Kumar H., Chand P., Jain S., S P. (2021). Lipid-Based Nanocarriers as Drug Delivery System and Its Applications. Nanopharmaceutical Advanced Delivery Systems.

[84] Kulbacka J., Pucek A., Kotulska M., Dubińska-Magiera M., Rossowska J., Rols M.P., Wilk K.A. (2016). Electroporation and Lipid Nanoparticles with Cyanine IR-780 and Flavonoids as Efficient Vectors to Enhanced Drug Delivery in Colon Cancer. Bioelectrochemistry.

[85] Talele P., Sahu S., Mishra A.K. (2018). Physicochemical Characterization of Solid Lipid Nanoparticles Comprised of Glycerol Monostearate and Bile Salts. Colloids Surf. B Biointerfaces.

[86] Gothwal A., Khan I., Gupta U. (2016). Polymeric Micelles: Recent Advancements in the Delivery of Anticancer Drugs. Pharm. Res..

[87] Wang J., Mongayt D., Torchilin V.P. (2005). Polymeric Micelles for Delivery of Poorly Soluble Drugs: Preparation and Anticancer Activity in Vitro of Paclitaxel Incorporated into Mixed Micelles Based on Poly(Ethylene Glycol)-Lipid Conjugate and Positively Charged Lipids. J. Drug Target..

[88] Chen L.F., Xu P.Y., Fu C.P., Kankala R.K., Chen A.Z., Wang S. (2020). Bin Fabrication of Supercritical Antisolvent (SAS) Process-Assisted Fisetin-Encapsulated Poly (Vinyl Pyrrolidone) (PVP) Nanocomposites for Improved Anticancer Therapy. Nanomaterials.

[89] Sari E.N., Soysal Y. (2020). Molecular and Therapeutic Effects of Fisetin Flavonoid in Diseases. J. Basic Clin. Health Sci..

[90] City of Hope Medical. Treatment of Frailty With Fisetin (TROFFi) in Breast Cancer Survivors. https://clinicaltrials.gov/ct2/show/NCT05595499..

[91] St. Jude Children’s Research Hospital An Open-Label Intervention Trial to Reduce Senescence and Improve Frailty in Adult Survivors of Childhood Cancer. https://clinicaltrials.gov/ct2/show/NCT04733534.

[92] Maher P. (2015). How Fisetin Reduces the Impact of Age and Disease on CNS Function. Front. Biosci.-Sch..

[93] Al-Ishaq R.K., Abotaleb M., Kubatka P., Kajo K., Büsselberg D. (2019). Flavonoids and Their Anti-Diabetic Effects: Cellular Mechanisms and Effects to Improve Blood Sugar Levels. Biomolecules.

[94] Croy S., Kwon G. (2006). Polymeric Micelles for Drug Delivery. Curr. Pharm. Des..

[95] Na N.-C., Dong C.-S., Jung M.-S. (2014). Composition Comprising Phenolic Compound for Preventing and Treating Liver Cirrhosis. WO Pantent.

[96] Cole G.M., Fruatshy S.A., Maher P., Schubert D. (2012). Medical Food for Cognitive Decline.

[97] Liping C., Shengnan J., Bingbing Z., Xiaodong X., Guoping D. Application of Fisetin in Inhibiting Proliferation of Pancreatic Cancer Cells and Mouse Pancreatic Cancer Tumors 2021.

[98] Park S.J., Kim K.H., Yoo Y.H. (2020). Method for Preparing Rhus Verniciflua Stokes Extract Containing Increased Fisetin Content, and Metastasis-Inhibiting Anticancer Agent Composition Containing Same.

[99] (2010). Hasan, Afaq Farrukh; Khan, Naghma; Mohammad, A.M. Methods of Treating Androgen Dependent Prostate Cancer By Administering an Active Pharmaceutical Ingredient Being Fisetin, 3,3′,4′,7-Tetrahydroxyflavone or a Derivative Thereof, in an Oral, Transdermal or Topical Dosage Form. U.S. Patent.

[100] Liping C., Jia S., Bingbing Z., Xiaodong X., Guoping D. Application of Fisetin in Combined Gemcitabine Pancreatic Cancer Treatment 2021.

[101] Sabarwal A., Dheeraj A., Singh R.P., Kaschula C.H. (2022). 4′-Substituted Analogues of Fisetin and Their Use in the Treatment of Cancer.

[102] MacDonald T., Kenney A.M., Dey A., Felker J. Methods of Treating Brain Cancer and Related Diagnostic Methods 2020.

[103] Cha G.J., Dong M.S., Jung N.C., Na C.S. (2002). Composition for Prevention and Treatment of Liver Cancer, Containing Phenolic Compound.

